# Astrocyte-to-neuron transportation of enhanced green fluorescent protein in cerebral cortex requires F-actin dependent tunneling nanotubes

**DOI:** 10.1038/s41598-021-96332-5

**Published:** 2021-08-18

**Authors:** Jing Chen, Junyan Cao

**Affiliations:** 1 Institute of Life Sciences, College of Life and Environmental Sciences, Hangzhou Normal University, Hangzhou, 311121 China; 2 Key Lab of Organ Development and Regeneration of Zhejiang Province, Hangzhou, Zhejiang China; 3 Key Lab of GEM Resource and Model Research of Hangzhou, Hangzhou, Zhejiang China

**Keywords:** Cell biology, Neuroscience

## Abstract

Tunneling nanotube (TNT), a dynamic cell–cell contact, is dependent on actin polymerization. TNTs are efficient in transporting ions, proteins and organelles intercellularly, which are important mechanisms in physiological and pathological processes. Reported studies on the existence and function of TNTs among neural cells focus on cultured cell for the convenience in detecting TNTs’ ultrastructure. In this study, the adeno-associated virus (AAV-*GFAP*-EGFP-p2A-cre) was injected into the cerebral cortex of knock-in mice ROSA26 GNZ. GFAP promoter initiated the expression of enhanced green fluorescent protein (EGFP) in infected astrocytes. At 10 days post injection (10 DPI), EGFP transferred from astrocytes in layer I–III to neurons in layer V. The dissemination of EGFP was not through endocytosis or exosome. Applying microscopes, we found that the intercellular transportation of EGFP through contact connection was F-actin dependent. Therefore, we concluded that EGFP transported from astrocytes to neurons in cortex via F-actin dependent TNTs. This study first proved that proteins transported intercellularly via TNTs in brain.

## Introduction

Tunneling nanotubes (TNT), a F-actin dependent principle of cell–cell contacted communication, was first visualized in mammalian cells by Rustom et al. in 2004^1^. It was then discovered to play important roles in different physiological and pathological processes in various cells in vitro^2^. In previous studies, TNT was found to transport mitochondria from progenitor/stem cells to rescue endothelial cell line—HUVEC^3,4^ and cardiomyoblast—H9c2^5^ from chemical induced injury and ischemia respectively. Moreover, TNT was discovered to transfer calcium flux^6,7^, organelles^8^ and cytosol^8,9^ to synchronize neighbor cells and induce cell differentiation. In pathological studies, TNT was proven to diffuse HIV among T cells^10^ and disseminate multi-drug resistance protein P-gp among breast cancer cells to acquire non-genetic resistance of chemotherapy^11^. In researches of neuron degeneration disease, TNT was demonstrated to spread prion^12,13^, mutant huntingtin^14,15^, amyloid β^16^ and Tau^17^ among neuronal cells, suggesting potential mechanisms in the deterioration of spongiform encephalopathies, Huntingtin’s disease and Alzheimer’s disease.

Astrocytes, an important regulator of neurite in the central nervous system, communicate intimately with neurons^18,19^. In vitro studies discovered that astrocytes can transmit calcium flux to neurons via TNT and induce depolarization of neurons^20^. Wang et al. demonstrated that developing neurons stretched filopodia to construct TNT contacting astrocytes with a long distance^20^. After examining the ultrastructure of TNT among mouse cathecholaminergic CAD cells and human neuroblastoma SH-SY5Y cells by cryo-transmission electron microscope, Sartori-Rupp et al. illustrated that most TNTs comprised of a bundle of individual TNTs (iTNTs). Each iTNT is filled by regularly organized bundles of F-actin^21^. However no in vivo study reported whether there was contact interaction between astrocytes and neurons yet. In this study, mediated by Adeno- associated virus (AAV), exogeneous enhanced green fluorescent protein (EGFP) was expressed specifically in astrocytes in mouse cortex. Surprisingly EGFP was transferred to neurons in layer V. Further we found that EGFP was not endocytosed into neurons. We hypothesized that EGFP transportation from astrocytes to neurons through TNT in cortex. This study provides first evidence for the transportation of proteins via contact communication between astrocytes and neurons in vivo.

## Results

### Astrocytes transferred EGFP to pyramidal neurons in the cortex

**Figure 1:**
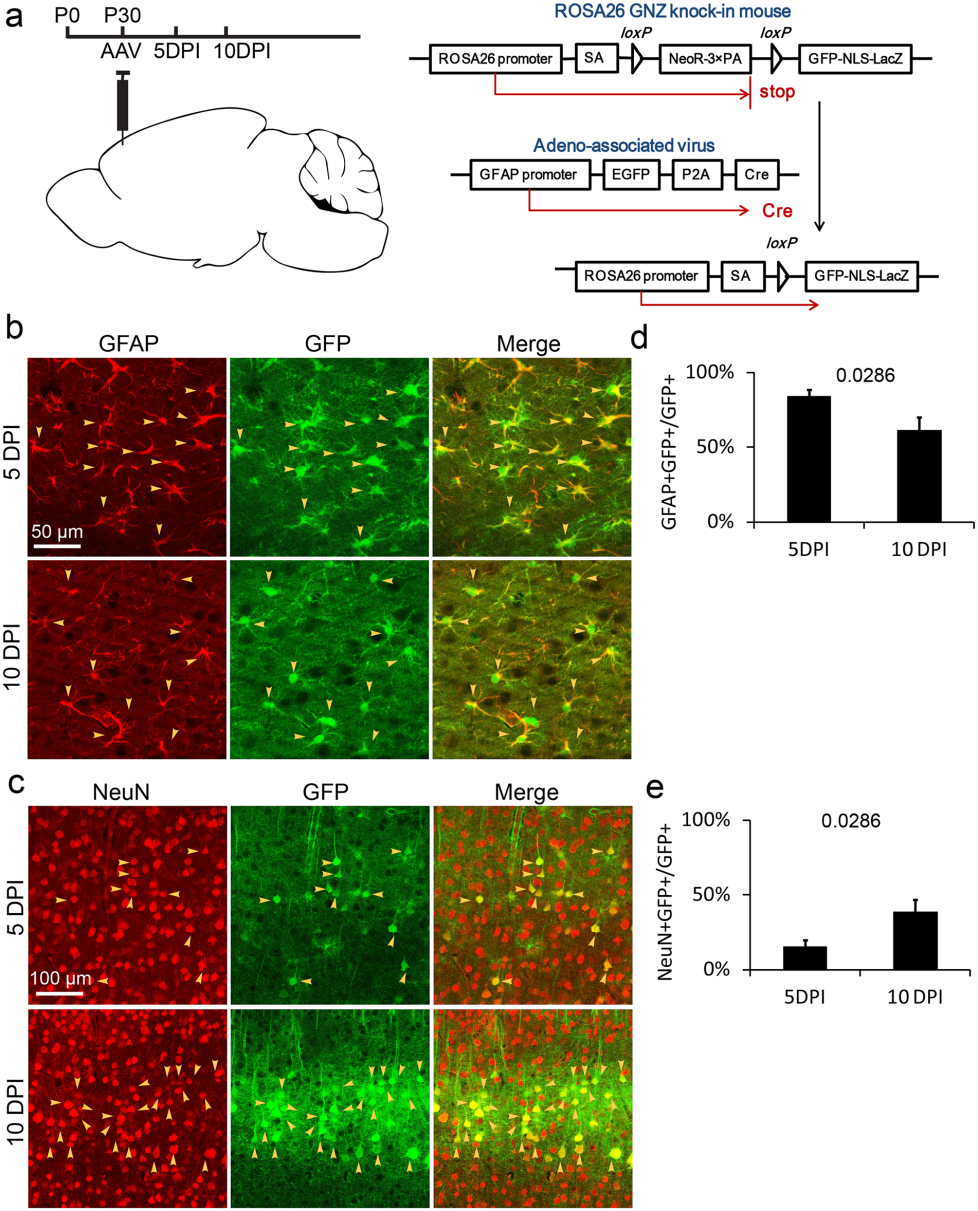
The existence of EGFP in the cortex. (**a**) Schematic illustration of stereotaxic injection of AAV-*GFAP*-EGFP-P2A-Cre in mouse cortex and the cre-loxP system in ROSA26 GNZ mice. AAV, Adeno-associated virus; 5 DPI, 5 days post injection of AAV; 10 DPI, 10 days post injection of AAV. (**b**) and (**c**) Detection of EGFP distribution in astrocytes of layer I–III and neurons of layer V 5 days and 10 days after injecting AAV. Yellow arrows, double positive cells. (**d**) and (**e**) The percentage of astrocytes and neurons in EGFP+ population at 5 DPI and 10 DPI. DPI, days post injection. Statistical analysis of significance was evaluated using unpaired two-tailed t-test, N = 3 independent mice.

**Figure 2:**
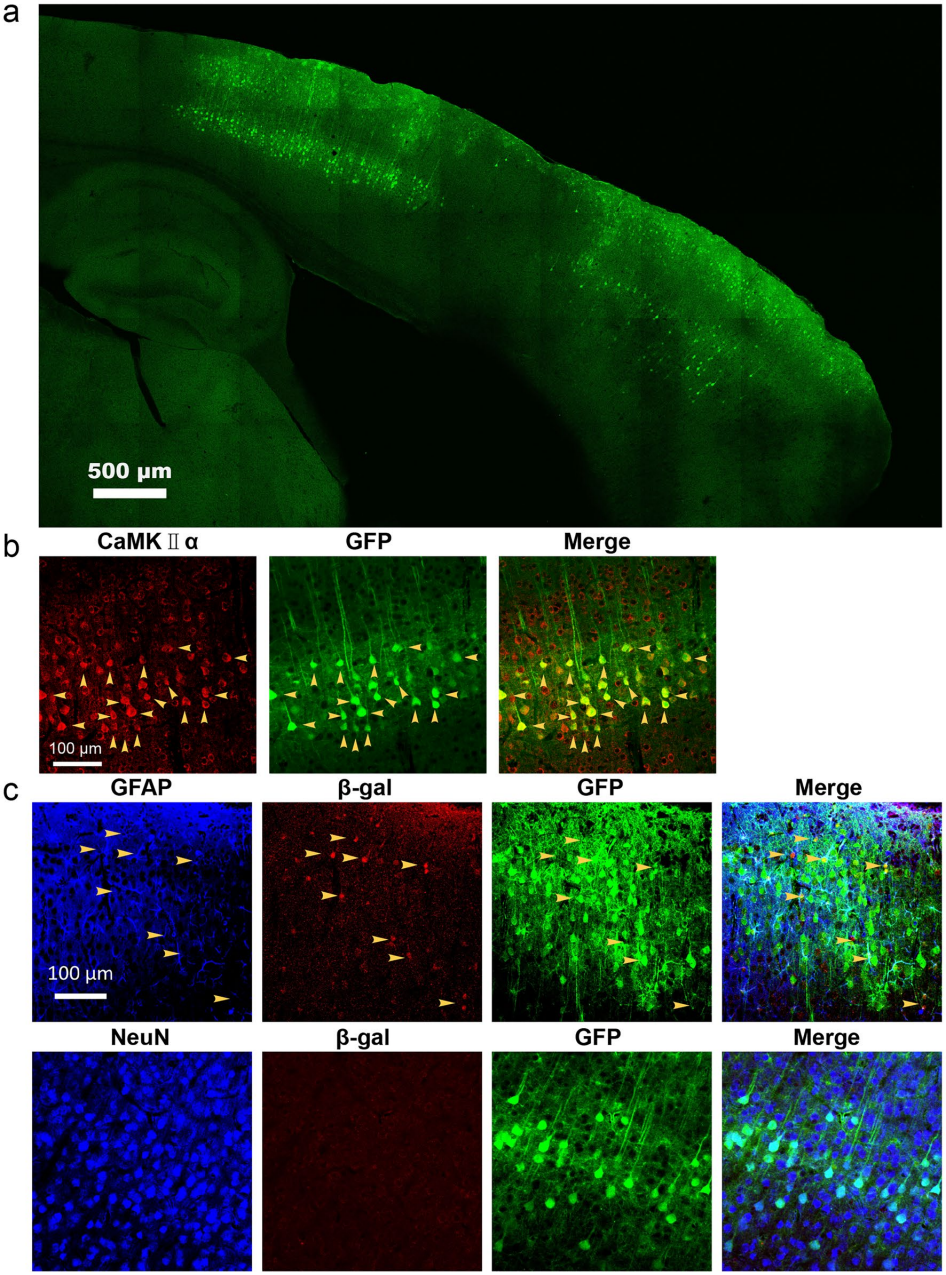
EGFP distribution in the cortex 10 days after injecting AAV. (**a**) The microscopic photo with low magnification shows EGFP+ cells in cerebral cortex. (**b**) Immunostaining of CaMK II α and EGFP to discriminate the subtype of EGFP+ neurons. Yellow arrows, double positive cells. (**c**) Immunostaining of GFAP/NeuN, β-gal and EGFP to discriminate cells expressing EGFP or receiving transported EGFP. Yellow arrows, triple positive cells.

AAV-*GFAP*-EGFP-P2A-Cre has been injected into the prefrontal cortex of homozygous ROSA26 GNZ knock-in mice with the age of P30 for 5–10 days (Fig. 1a). The expression of cre and EGFP are initiated by promoter *GFAP* in GFAP + cells. Recombinase cre recognizes and cuts the sequence of lox p-stop codon-lox p in transgenic mouse ROSA26 GNZ, and then induces the expression of GFP-β-galactosidase fusion protein with a nuclear positioning sequence (Fig. 1a)^22^. After 5 days (5 DPI), EGFP existed in both astrocytes and neurons in the cortex with proportions of 84.3 ± 4% and 15.7 ± 4% respectively (Supplementary Fig. 1, Fig. 1b–e). After 10 days (10 DPI), EGFP was also detected in both astrocytes and neurons, and in the population of EGFP+ cells, 61.5 ± 8.6% were astrocytes and 38.5 ± 8.6% were neurons (Supplementary Fig. 1, Fig. 1b–e). The proportion of EGFP+ neurons increased significantly from 10 to 5 DPI (Fig. 1d,e). According to cell density of the cortex shown by DAPI staining^23^, EGFP+ astrocytes were mostly in layer I–III and EGFP+ neurons were predominantly in layer V (Fig. 2a, Supplementary Fig. 2). All of the EGFP+ neurons were CaMK II α positive (Fig. 2b), indicating that they are pyramidal neurons. To confirm the specificity of promoter—*GFAP*, we detected the expression of nuclear localized β-galactosidase induced by Cre. Results indicated that β-galactosidase expressed specifically in astrocytes (Fig. 2c). Since the expression of Cre is initiated by promoter—*GFAP*, the result demonstrated that promoter—*GFAP* is an astrocyte specific promoter. In this study, a *P2A* sequence departs products of recombinant gene *EGFP* and *cre* carried by adeno-associated virus (Fig. 1a). According to Le et al.’s study, cre recombinase is a nuclear localizing protein both in free state and in cre-EGFP fusion state^24^. The result of this study that neurons did not express β-galactosidase (Fig. 2c), demonstrated that cre recombinase expressed in astrocytes could not be transferred to neurons, which re-proved the nuclear localization property of cre. In conclusion, results demonstrated that EGFP in neurons was not expressed in situ but transported from astrocytes.

### EGFP was not transferred intercellularly via endocytosis

**Figure 3:**
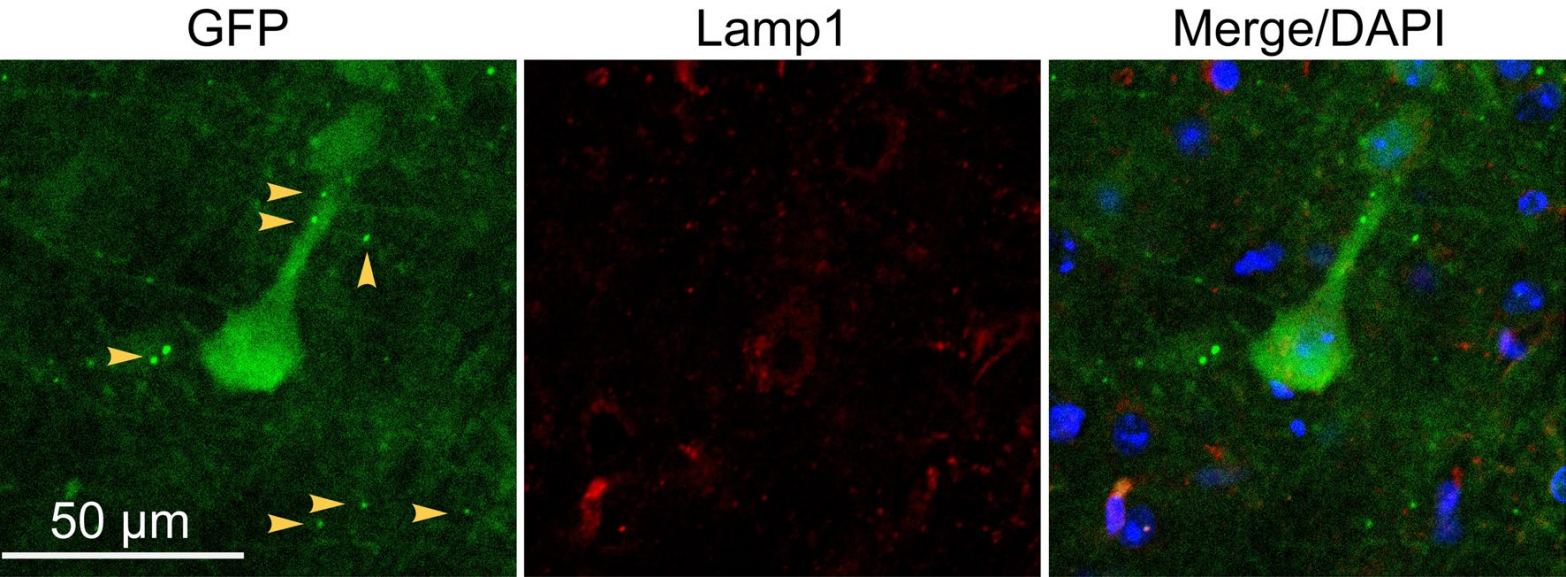
Immunostaning of lamp1 and EGFP to demonstrate the localization of endocytotic vesicles and EGFP particles in the cortex. Yellow arrows, EGFP particles do not co-localize with lamp1.

Most EGFP+ neurons existed on layer V and are not close to EGFP expressing astrocytes in layer I–III of the cortex (Fig. 2a, Supplementary Fig. 2). One possible way of transferring EGFP from astrocytes to neurons is secretion. If EGFP was secreted as a free molecule to the extracellular environment, it might be endocytosed by the target cell and then fused with lysosome. To detect whether EGFP was transferred from astrocytes to neurons through exocytosis and endocytosis, we examined whether lysosome marker—lamp1^25^ was co-localized with EGFP. The result demonstrated that EGFP particles were not localized with lamp1 (Fig. 3), indicating free EGFP was not secreted from astrocytes to extracellular environment and enter neurons through endocytosis.

**Figure 4:**
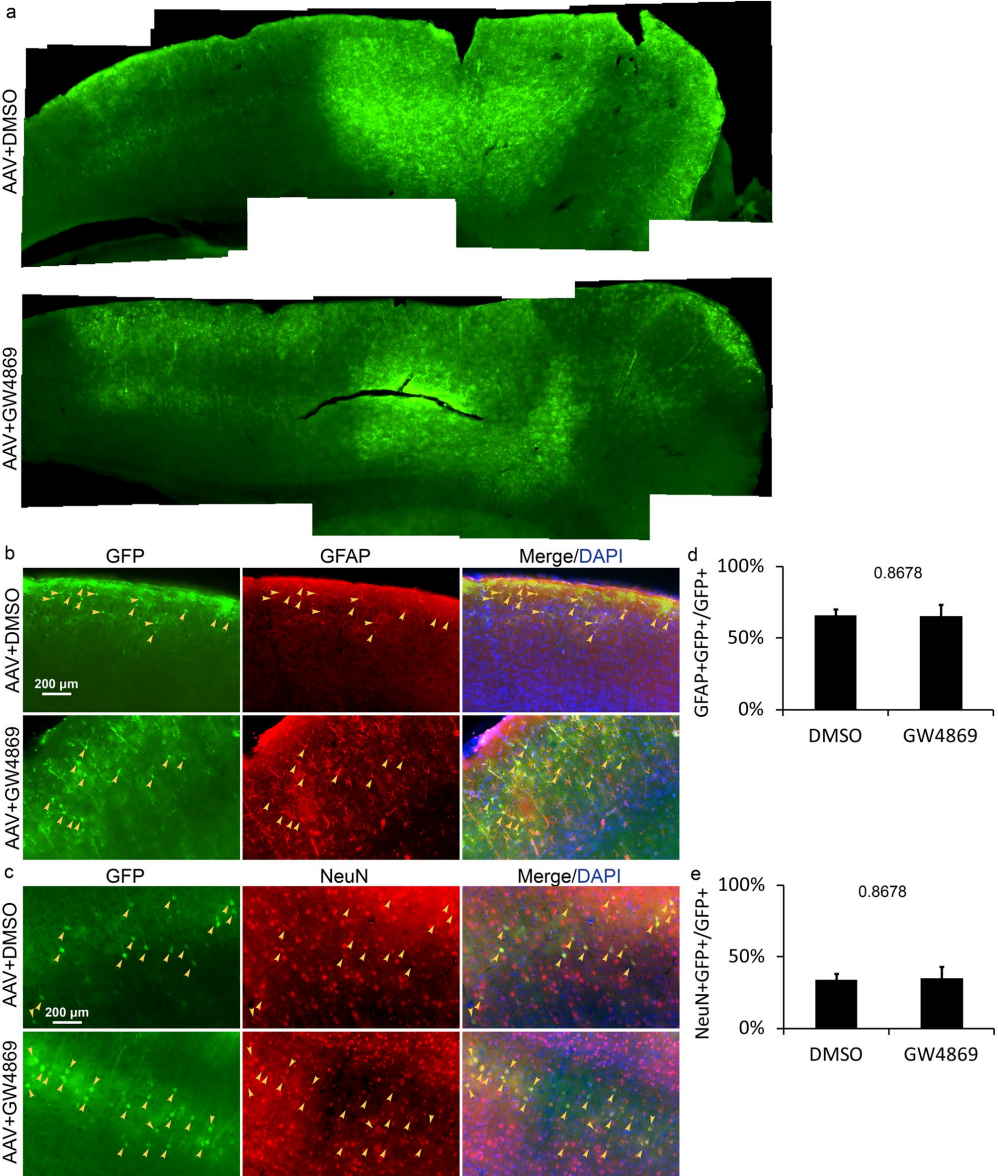
EFGP distribution in the cortex 10 days after injecting AAV and DMSO/GW4869. (**a**) The microscopic photo with low magnification shows EGFP+ cells in cerebral cortex. (**b**) and (**c**) Detection of EGFP distribution in astrocytes of layer I–III and neurons of layer V at 10 DPI. Yellow arrows, double positive cells. (**d**) and (**e**) The percentage of astrocytes and neurons in EGFP+ population at 10 DPI. Statistical analysis of significance was evaluated using unpaired two-tailed t test, N = 3 independent mice.

However, EGFP might be transported by nanovesicles with membrane—exosomes. The exosome is an efficient vector to cargo biomolecules and transmit signals intercellularly in the neuron-astrocytes network^26^. GW4869—an inhibitor of neutral sphingomyelinase can block the biogenesis and release of exosomes efficiently^27^. Therefore, the extent of GW4869 prohibiting intercellular spreading of EGFP manifests the reliance that EGFP transportation places on exosomes. To confirm whether EGFP was transferred from astrocytes to neurons via exosomes, we inject GW4869 in the same site injected with AAV-*GFAP*-Cre-EGFP and examine the dissemination of EGFP. Previous studies demonstrated that 10–20 μM GW4869 could decrease more than 70% exosomes released from neurons^28^ and astrocytes^29^. Here we injected 1 μl of 80 μM GW4869 in the cortex to inhibit exosome formation thoroughly. To avoid the influence of GW4869 on the process of AAVs infecting cells, GW4869 was injected in the prefrontal cortex 30 min after injection of AAV-*GFAP*-EGFP-P2A-Cre, and the control group was injected with 0.5% (volume ratio) DMSO in PBS 30 min after the injection of AAV-*GFAP*-EGFP-P2A-Cre. After 10 days, we examined the existence of EGFP in the brain, and found that GW4869 did not affect the distribution of EGFP in the cortex (Fig. 4a). The results showed that EGFP existed in astrocytes and neurons with the proportion of 65.1 ± 8.4% and 34.9 ± 8.4% respectively (Fig. 4b–e), which is not significantly different from the control with 66.1 ± 4.3% astrocytes and 33.9 ± 4.3% neurons in EGFP+ population (Fig. 4b–e), indicating that EGFP was not transported intercellularly through exosomes.

### EGFP was transferred between astrocytes and neurons via TNT

TNTs are membrane processes connecting two cells and open at both ends, which makes it a highway to transport ions, molecules and organelles efficiently and specifically^2,30^. The TNT is a potential way of the intercellular transportation of EGFP.

In vitro studies demonstrated that each TNT comprises of 2–11 iTNTs. The diameter of iTNT ranges from 50 to 200 nm. Adding the space between iTNTs, the diameter of TNTs ranges from 145 to 700 nm^1,21^. Furthermore, TNTs are transient intercellular connecting structure which lasts for 10–15 min^20^. As a fragile, tiny and dynamic ultrastructure, the TNT is susceptible to chemicals and the changing environment^31^ in which we prepare experimental tissue samples. Since markers used to detect TNTs, such as membrane marker—wheat germ agglutinin and F-actin binding molecule—phalloidin^1^ are not TNTs specific but ubiquitous in membrane processes of various cells, the background is too noisy to discriminate TNTs in solid tissues. All in all, TNTs are hardly detected in solid tissues under microscopes, which might explain in vivo study on TNTs in brain was seldom reported.

**Figure 5:**
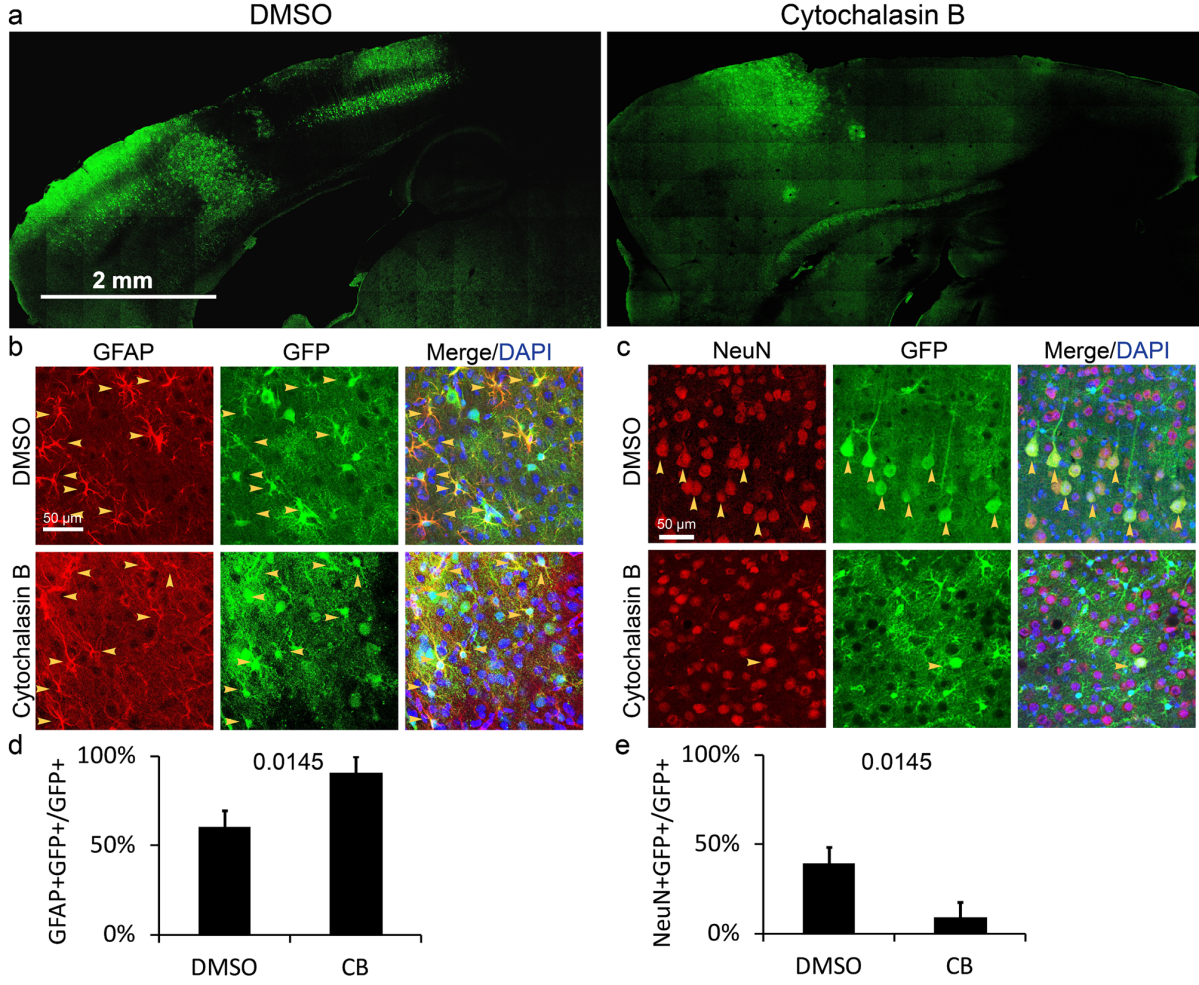
EFGP distribution in the cortex 10 days after injecting AAV and DMSO/cytochalasin B. (**a**) The microscopic photo with low magnification shows EGFP+ cells in cerebral cortex. (**b**) and (**c**) Detection of EGFP distribution in astrocytes of layer I–III and neurons of layer V at 10 DPI. Yellow arrows, double positive cells. (**d**) and (**e**) The percentage of astrocytes and neurons in EGFP+ population at 10 DPI. CB, cytochalasin B. Statistical analysis of significance was evaluated using unpaired two-tailed t test, N = 3 independent mice.

F-actin is a crucial skeleton to sustain the structure of TNTs^20,21^. To examine whether intercellular transferring EGFP was TNTs dependent, an inhibitor of F-actin assembly—cytochalasin B^32^ was applied in the in vivo study. According to the kinetics study of F-actin, around 1 μg/ml cytochalasin B could reduce 90% polymerization^32^. Studies on mammal cells found that less than 0.2 μg/ml cytochalasin B can inhibit 90% intercellular TNTs^33^. Here we used 1 μl of 50 μg/ml cytochalasin B to prohibit TNTs in cortex thoroughly. To avoid the influence of cytochalasin B on the process of AAVs infecting cells, cytochalasin B was injected to the prefrontal cortex 30 min after the injection of AAV-*GFAP*-EGFP-P2A-Cre, and the control group was injected with 1 μl of 1% (volume ratio) DMSO in PBS 30 min after the injection of AAV-*GFAP*-EGFP-P2A-Cre. 10 days later, the existence of EGFP in the brain was examined. With the treatment of cytochalasin B, the distribution of EGFP in the cortex was obviously differently from the control (Fig. 5a). The proportion of astrocytes and neurons in EGFP+ cells were 91 ± 8.8% and 9 ± 8.8% respectively in cytochalasin B treated group (Fig. 5b–e), and the proportion of astrocytes and neurons in EGFP+ cells were 60.6 ± 9.2% and 39.4 ± 9.2% respectively in the control group (Fig. 5b–e). According to the statistical analysis, the population of EGFP+ neurons decreased significantly after the treatment of cytochalasin B (Fig. 5d,e). Exosome secretion is also regulated by F-actin ^34^, but exosome specific inhibitor GW4869 did not block the transferring of EGFP (Fig. 4a–e), which indicating that the exosome is not critical to astrocyte-to-neuron transportation of EGFP. Since cytochalasin B destroyed EGFP transportation drastically, the result suggested that EGFP transportation from astrocytes to neurons in the cortex mainly relyed on F-actin dependent TNTs.

**Figure 6:**
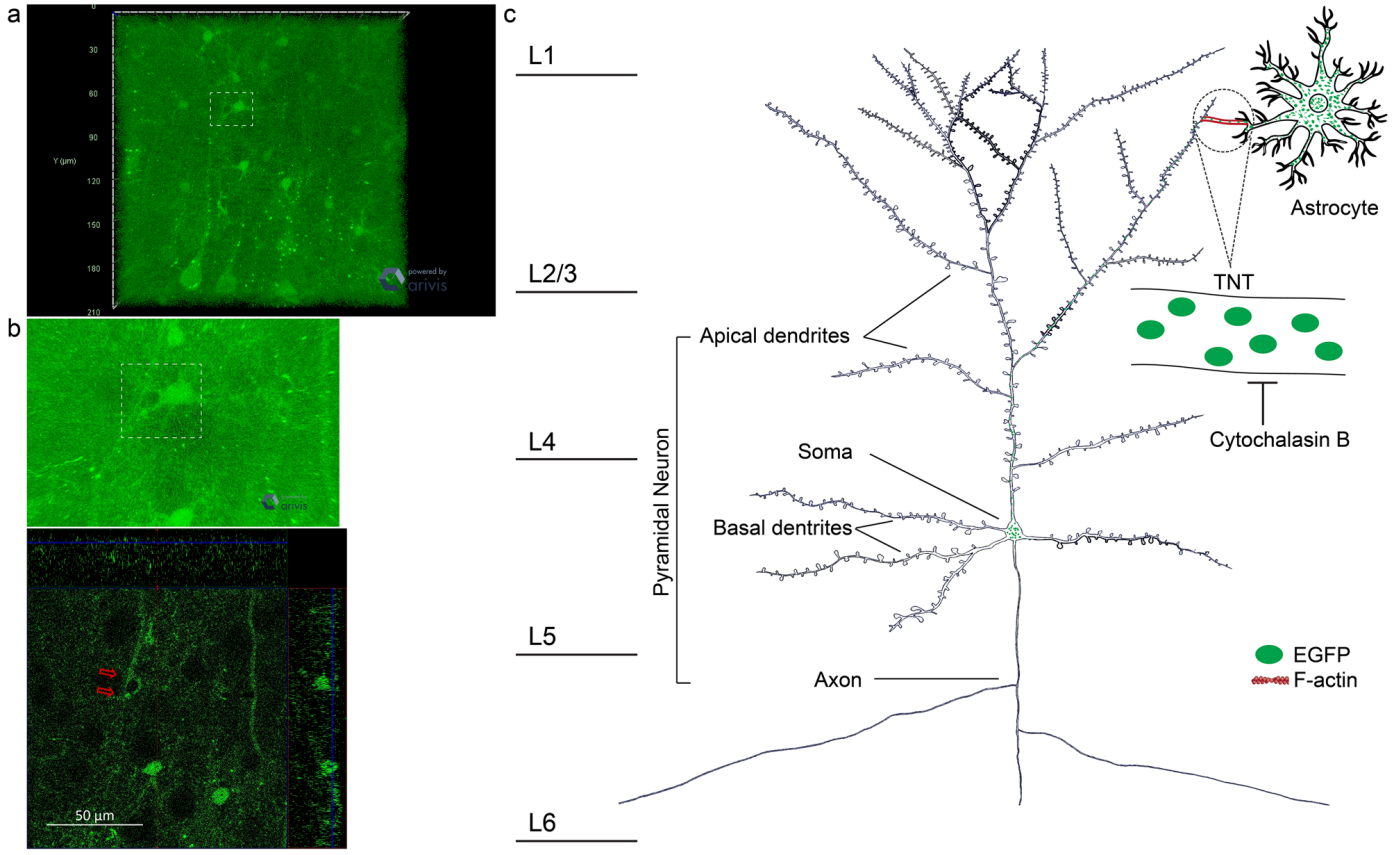
Illustration of the working model for EGFP transportation from astrocytes to neurons in cerebral cortex. (**a**) Detection of the contact connection between an apical dendrite and an astrocyte process by confocal microscopy. (**b**) High magnified stereophotograph (above) and 2D image (below) of the contact connection. (**c**) Schematic illustration: Astrocytes in layer I–III express EGFP. Pyramidal neurons in layer V stretch out apical dendrites to layer I–III with branches close to EGFP expressing astrocytes. With the assistance of F-actin which is enriched in sprouting dendrite branches, pyramidal neurons develop tunneling nano-tubules to astrocytes. EGFP disseminates from astrocytes to neurons through the tunneling nano-tubule. Red arrows, TNT. TNT, tunneling nano-tubule.

By capturing the stereo photograph of cerebral cortex, we found that a neuron’s apical dendrite and two astrocyte’s processes were physically connected (Fig. 6a,b), which contained TNTs transporting EGFP particles. To visualize the ultrastructure of TNTs, immunoelectron microscopy (IEM) was applied to detect the distribution of EGFP in the cortex. In IEM photos, it was not found that EGFP existed in membrane vesicles such as lysosomes and exosomes (Supplementary Fig. 3). Therefore, we believe that EGFP was not endocytosed into neurons. A tunnel connecting an astrocyte and a neuron’s dendrite with EGFP was a TNT with a diameter around 30 nm (Supplementary Fig. 3). In conclusion, all results demonstrated that EGFP was transported from astrocytes to neurons via F-actin dependent TNT (Fig. 6c).

## Discussion

Astrocytes are crucial elements in the nervous system to provide structural and metabolic support for the activity and homeostasis of neurons^35^. As far as we know, astrocytes and neurons exchange neurotransmitters and metabolic molecules through particular membrane channels^36,37^. Astrocytes were also reported to transport proteins, such as apolipoprotein D to neurons through exosomes^38^. In vitro studies demonstrated that astrocytes transferred prions^12^ and calcium ions^20^ to neurons via TNT, unveiling a highway for specific intercellular transportation between astrocytes and neurons. However, no in vivo study reports TNT between astrocytes and neurons yet. Here we discovered that EGFP produced in astrocytes of cortex layer I–III were transferred specifically and efficiently to neurons in layer V through contact connection (Figs. 2, 6, Supplementary Fig. 2, Supplementary Fig. 3). The process of the intercellular transportation is F-actin dependent (Fig. 5) and endocytosis/exosome independent (Figs. 3, 4), indicating that EGFP transferred from astrocytes to neurons via TNT. This study firstly provides in vivo evidence of intercellular transportation of proteins via TNTs in the nervous system, and experimental methods to study TNTs in solid tissues.

According to reported studies, TNTs between cultured cells were conveniently detected with optical microscopes and electron microscopes. The average diameter of iTNT is around 100 nm, and a TNT is composed of more than 2 iTNTs^1,21^. In this study, the contact connection between astrocyte processes and dendrites of pyramidal neurons was detectable, but it is hardly to detect the structure of TNTs in the cortex with optical microscopes (Fig. 6a). Applying transmission electron microscopy, we found that the diameter of TNTs in vivo was less than the reported diameter in vitro, and the assembly of iTNTs bundles was not obvious (Supplementary Fig. 3). Therefore, the characteristics of TNTs in solid tissues are different from those in cultured condition.

TNTs are contact passages of nearby cells. In the cortex, GFP was transferred from astrocytes in layer I–III to neurons in layer V. Although somas of both cells are distant, membrane processes, such as apical dendrites of neurons, can extend near astrocytes in layer I–IV^39^(Fig. 6). According to Wang et al.’s study, in vitro developing neurons stretched out processes to distant astrocytes to form TNTs, which is microtubules and F-actin dependent^20^. Since directed dendrite growth requires dynamic F-actin population stalling at the branching sites^40,41^, we hypothesize that TNTs in the cortex were formed between apical dendrites and astrocyte somas/processes with a close distance (Fig. 6, Supplementary Fig. 3). Wang et al. discovered that connexin 43 is an important inducer of TNT-like ultrastructure to connect premature neurons and astrocytes which transfer Ca2+ signals between them^20^, suggesting that TNTs form in the base of the gap junction. In this study, immunofluorescence assays were applied to detect whether connexin 43 involved in the construction of TNTs, and a polypeptide binder of connexin 43—gap 26 was applied to block the formation of gap junction to explore the function of connexin 43 in TNT-dependent intercellular transportation. Results demonstrated that connexin 43 did not participate in the construction of astrocyte-to-neuron TNTs (Supplementary Fig. 4) and intercellular transportation of EGFP (Supplementary Fig. 5) in the cortex. Given that each gap junction protein can form homotypic or hetergenic channels with specific gap junction proteins^42^, TNTs might form specifically between astrocytes and pyramidal neurons in the cortex for their expression of particular gap junction proteins except connexin 43, which might explain the targeted dissemination of EGFP from astrocytes in layer I–III to neurons in layer V.

β-Amyloid(1–42) (Aβ42), a major component of amyloid plaques, accumulates within pyramidal neurons in the brains of individuals with Alzheimer’s disease (AD) and Down syndrome. Nagele et al.^43^ found that besides pyramidal neurons, Aβ42 accumulated in astrocytes of cortical molecular layer (layer I), which showed moderate to advanced AD pathology. How Aβ42 transferred from neurons to astrocytes in vivo is unclear yet. In this study, we found that fluorescent proteins were transported between astrocytes in layer I–III and pyramidal neurons in layer V via TNTs. Considering Wang et al.’s^16^ study reported that Aβ42 was transferred among astrocytes via TNTs in vitro, the conclusion of our study that TNTs formed between pyramidal neurons and astrocytes might explain the mechanism of Aβ42 dissemination from pyramidal neurons to astrocytes and the exacerbation of AD pathology. Prion, an infectious protein, can spread intercellularly in the central nervous system. The aggregation of prion in the neuron induces neuron degeneration causing spongiform encephalopathies. In that case, the mechanism of intercellular transmission of prion is a potential target to cure spongiform encephalopathies. In vitro studies discovered that prion can transfer from astrocytes to neurons and among neurons via TNTs^12^. This study provides an in vivo model to study the intercellular transmission of prion. Furthermore, in vitro studies, prion like proteins such as mutant huntingtin^15^ and pathological Tau^17^ were found to disseminate among neurons or astrocytes through TNTs, suggesting they might be transported among astrocytes and neurons in the cortex via TNTs like EGFP. In conclusion, results of our study provide experimental evidences for pathological studies of neuron degeneration diseases.

## Materials and methods

### Animal use statement

Animal experiments were conducted in compliance with the ARRIVE guidelines 2.0., and were approved by the Institutional Animal Care and Use Committee of the Hangzhou Normal University. According to AVMA Guidelines for the Euthanasia of Animals (2020), animals were anesthetized by Avertin (i.p.) in experiments. All methods were carried out in accordance with relevant guidelines and regulations.

### Animals

Homozygous ROSA26 GNZ knock-in mice were from the Jackson Laboratory (stock #008,606). Primers OIMR8038 (5′-TAAGCCTGCCCAGAAGACTC-3′), OIMR8545 (5′-AAAGTCGCTCTGAGTTGTTAT-3′) and OIMR9539 (5′-TCCAGTTCAACATCAGCCGCTACA-3′) with a 575 bp PCR product were used for genotyping of ROSA26 GNZ^22^. Unless indicated, mice were housed in a room with a 12-h light/dark cycle with access to food and water ad libitum. Homozygous ROSA26 GNZ mice with either sex were used for experiments^44^.

### Antibodies and reagents

Information on commercial mouse antibodies is as follows: GFAP (MAB360, 1:500 for IF) and NeuN (MAB377, 1:500 for IF) were from Millipore (Temecula, CA, USA). Information on commercial rabbit antibodies is as follows: GFP (TP401, 1:500 for IF) was from Torrey Pines Biolabs Inc. (Secaucus, NJ, USA); CaMK II α (bs-0564R, 1:200 for IF) and Connexin 43 (bs-0651R, 1:100 for IF) were from BIOSS Antibodies (Beijing, China). Information on commercial chicken antibodies is as follows: β-galatosidase (ab9361, 1:500 for IF) was from Abcam (Cambridge, MA, USA). Information on commercial rat antibodies is as follows: Lamp1 (ab25245, 1:500 for IF) was from Abcam (Cambridge, MA, USA). Information on guinea pig antibodies is as follows: EGFP (1:500 for IF) was from Dr. Hao Huang in Hangzhou Normal University. Information on second antibodies is as follows: Goat anti-Mouse IgG (H + L) Cross-Absorbed Secondary Antibody, Alexa Fluor 405 (A31553, 1:200 for IF), Donkey anti-Rat IgG (H + L) Highly Cross-Absorbed Secondary Antibody, Alexa Fluor 594 (A21209, 1:1000 for IF), Goat Anti-Guinea Pig IgG (H + L) Highly Cross-Absorbed Secondary Antibody, Alexa Fluor 488 (A11073, 1:500 for IF) and Donkey anti-Rabbit IgG (H + L) Cross-Absorbed Secondary Antibody, Alexa Fluor 488 (A21206, 1:1000 for IF) were from Invitrogen (Rockford, IL, USA); Goat anti-chicken IgY, Alexa Fluor 594 (ab150176, 1:500 for IF) was from Abcam; Nanogold-IgG goat anti rabbit (2003, 1:50 for IEM) was from Nanoprobes (Yaphank, NY, USA).

The inhibitor of exosome biogenesis/release—GW4869 (D1692) was purchased from Sigma-Aldrich (Shanghai, China). GW4869 was dissolved in DMSO (Sigma-Aldrich, Shanghai, China) to the stock concentration of 16 mM, and then was diluted with 0.9% NaCl solution to the working concentration of 80 μM. The inhibitor of TNT formation—cytochalasin B (250233) was purchased from Sigma-Aldrich (Shanghai, China). Cytochalasin B was dissolved in DMSO to the stock concentration of 50 mg/ml, and then was diluted with 0.9% NaCl solution to the working concentration of 50 μg/ml. The inhibitor of connexin 43 function—gap 26 (T5192) was purchased from TargetMol (Shanghai, China). Gap 26 was dissolved in ddH_2_O to the working/stocking concentration of 1 mg/ml.

### Stereotaxic injection of AAV viruses and chemicals

AAV-*GFAP*-EGFP-P2A-Cre plasmids were constructed by standard methods, packaged as AAV2/8 viruses, and produced with titers of 1 × 10^12^ particles per ml by OBio (Shanghai, China). As described previously^44^, mice were anesthetized by 1.2% Avertin (0.2 ml/10 g, i.p.) and mounted at stereotaxic apparatus (RWD68025, RWD, Shenzhen, China). AAV-*GFAP*-EGFP-P2A-Cre (1 μl) was injected into the cortex (from bregma in mm, cortex, M-L: ± 1.0, A-P: − 1.5, D-V: 1.0) under control of micropump (#53311, Stoeling Co., Wood Dale, IL, USA) at speed of 0.5 μl/min. Injecting needles (Hamilton NDL ga33/30 mm/pst4, Switzerland) were withdrawn 5 min after injection. To observe the influence of chemicals—GW4869, cytochalasin B and gap 26 on the transportation of EGFP, 1 μl of 80 μM GW4869, 1 μl of 50 μg/ml cytochalasin B, 1 μl of 1 mg/ml gap 26, 1 μl of 0.5% DMSO or 1 μl of 1% DMSO was injected into the cortex (from bregma in mm, cortex, M-L: ± 1.0, A-P: − 1.5, D-V: 1.0) under the control of micropump at speed of 0.5 μl/min 30 min after the injection of AAV viruses. Injecting needles were withdrawn 5 min after injection.

### Immunofluorescence staining

Mice were anesthetized by 1.2% Avertin (0.2 ml/10 g, i.p.) and were perfused with 4% paraformaldehyde in 0.9% (w/v) NaCl. Mouse brains were isolated and fixed in 4% PFA overnight, and then washed with 0.01 M PBS (KH_2_PO_4_ 2 mM, Na_2_HPO_4_ 8 mM, NaCl 136 mM, KCl 2.6 mM, pH 7.4) twice. The fixed brains were kept in 0.01 M PBS with 1% ProClin 200 in 4 °C until sectioned by vibrating microtome (Leica VT1000S). Soft agar-embedded mouse brains were cut into 50 μm sections and subjected to immunostaining. As described previously^44^, brain slices were incubated with blocking buffer (10% fetal bovine serum and 0.1% TritonX-100 in 0.01 M PBS) for 1 h at room temperature, and then incubated at 4 °C overnight with primary antibodies diluted in blocking buffer. After being washed three times with PBS, samples were incubated at room temperature for 1 h with secondary antibodies, and then washed and mounted on adhesion microscope slides (CITOTEST) with fluorescent mounting medium (0.5% N-propyl gallate, 50% glyceral in 20 mM Tris, PH 8.0). To analysis the ratio of GFAP + cells and NeuN + cells in the population of GFP + cells, all GFP + cells were counted in the cortex of 1–3 slices of each mouse. Results of 3 mice in each experimental group were analyzed statistically. Images were taken by a Zeiss LSM710 confocal microscope and Nikon ECLIPSE Ti with exactly same scanning conditions for paired experiments. Brightness/contrast of images was adjusted by Zen (Zeiss) and NIS-Elements BR (Nikon). Analysis was run by Image J.

### Immunoelectron microscopy

Mice were anesthetized by 1.2% Avertin (0.2 ml/10 g, i.p.) and were perfused with 4% paraformaldehyde and 0.5% glutaraldehyde in 0.1 M PB (NaH_2_PO_4_*H_2_O 0.023 M, Na_2_HPO_4_ 0.077 M, pH 7.4). Mouse brains were isolated and fixed in perfusion solution overnight. The fixed brains were cut into 50 μm sections by vibrating microtome and subjected to immunostaining. Brain slices were kept and re-fixed in perfusion solution for 2 h. Following washes with 0.1 M PB (3 × 15 min), samples were incubated with glycine solution (50 mM glycine in 0.1 M PB) for 30 min and then were washed with 0.1 M PB for 15 min. Samples were incubated in 0.1 M PB containing 0.05% triton X-100 for 15 min. After being washed with 0.1 M PB for 15 min, samples were incubated with blocking buffer (0.1% BSA-cTM in 0.1 M PB) for 30 min and then were incubated with the primary antibody overnight at 4 °C. Following washes with blocking buffer (6 × 10 min/time), samples were incubated with the second antibody for 1 h at room temperature and then overnight at 4 °C. Following washes with blocking buffer (6 × 10 min/time) and 0.1 M PB (2 × 10 min/time), samples were re-fixed with 2.5% glutaraldehyde for 2 h at room temperature. Following washes with 0.1 M PB (3 × 10 min/time), ddH_2_O (6 × 5 min/time), and 0.02 M sodium citrate buffer (pH 7.0) (3 × 5 min/time), samples were floated in freshly mixed enhancement solution (2114, Nanoprobes) for 6–8 min, and then were rinsed with ddH_2_O and 0.1 M PB. Samples were incubated in 1% OsO_4_ for 30 min. After washes with ddH_2_O (3 × 5 min/time), samples were incubated with 2% uranium acetate for 30 min^45^. After washes with ddH_2_O (3 × 5 min/time), samples were dehydrated with alcohol and acetone and then embedded in Epon (Electron Microscopy Sciences, Hatfield, PA). Images were taken by FEI Tecnai G2 Spirit Bio TWIN.

### Statistical analysis

All data were expressed as the mean ± the standard deviation from at least three independent experiments and analyzed using unpaired two tailed Student's t‑test by Excel 2007 (Microsoft, Redmond, Washington, USA). *P* < 0.05 was considered to indicate a statistically significant difference.

## Supplementary Information

Supplementary Information 1.

Supplementary Information 2.

Supplementary Information 3.

Supplementary Information 4.

Supplementary Information 5.

Supplementary Information 6.

## Data Availability

The data that support the findings of this study are available from the corresponding author upon request.
