# Supplementary figures and images for: Astrocyte-to-neuron transportation of enhanced green fluorescent protein in cerebral cortex requires F-actin dependent tunneling nanotubes
Source: Sci Rep. 2021 Aug 18;11:16798. doi: 10.1038/s41598-021-96332-5 (PMC8373867; doi:10.1038/s41598-021-96332-5)

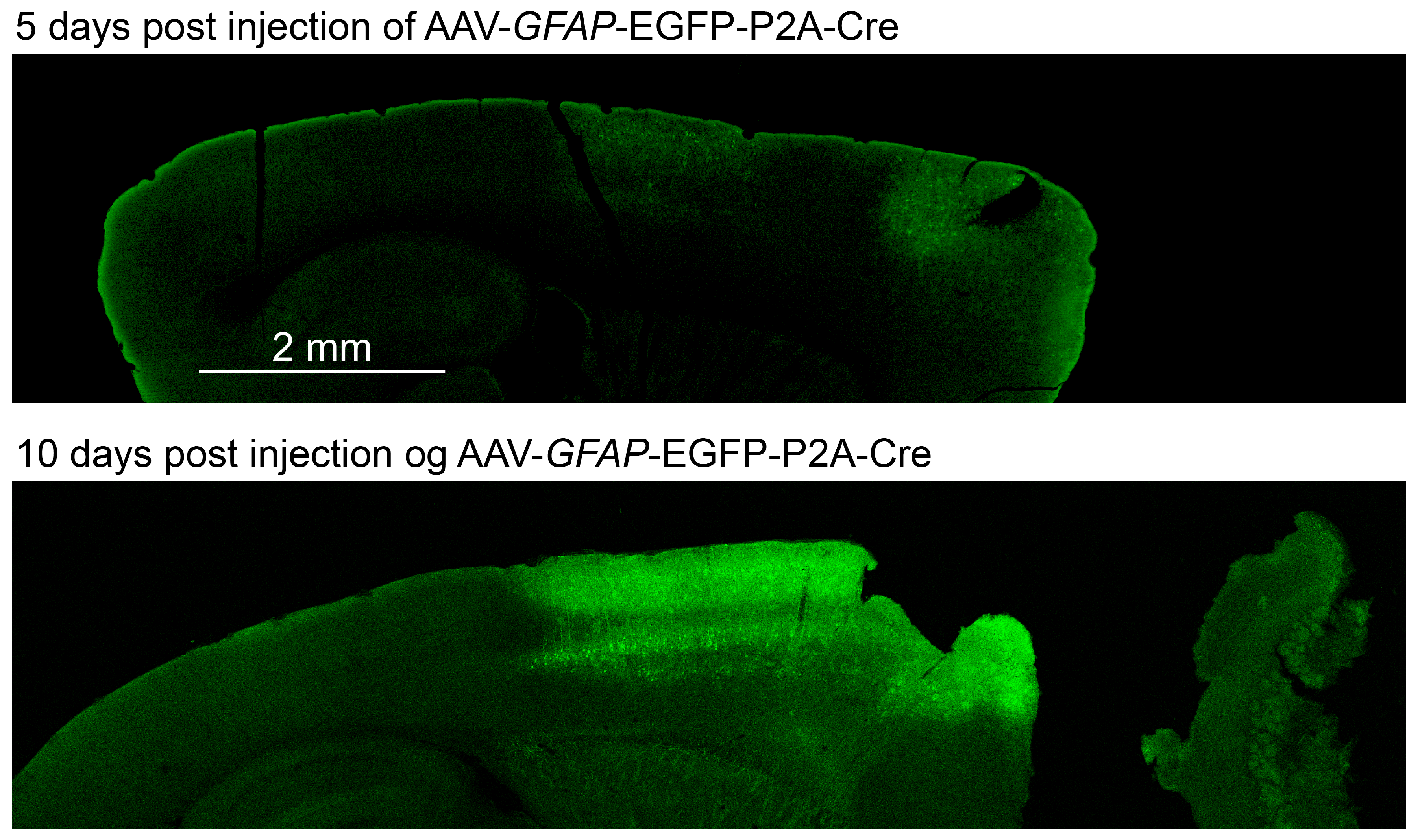

Supplement: Supplementary file 1 — Supplementary Information 1. [file 41598_2021_96332_MOESM1_ESM.tif]

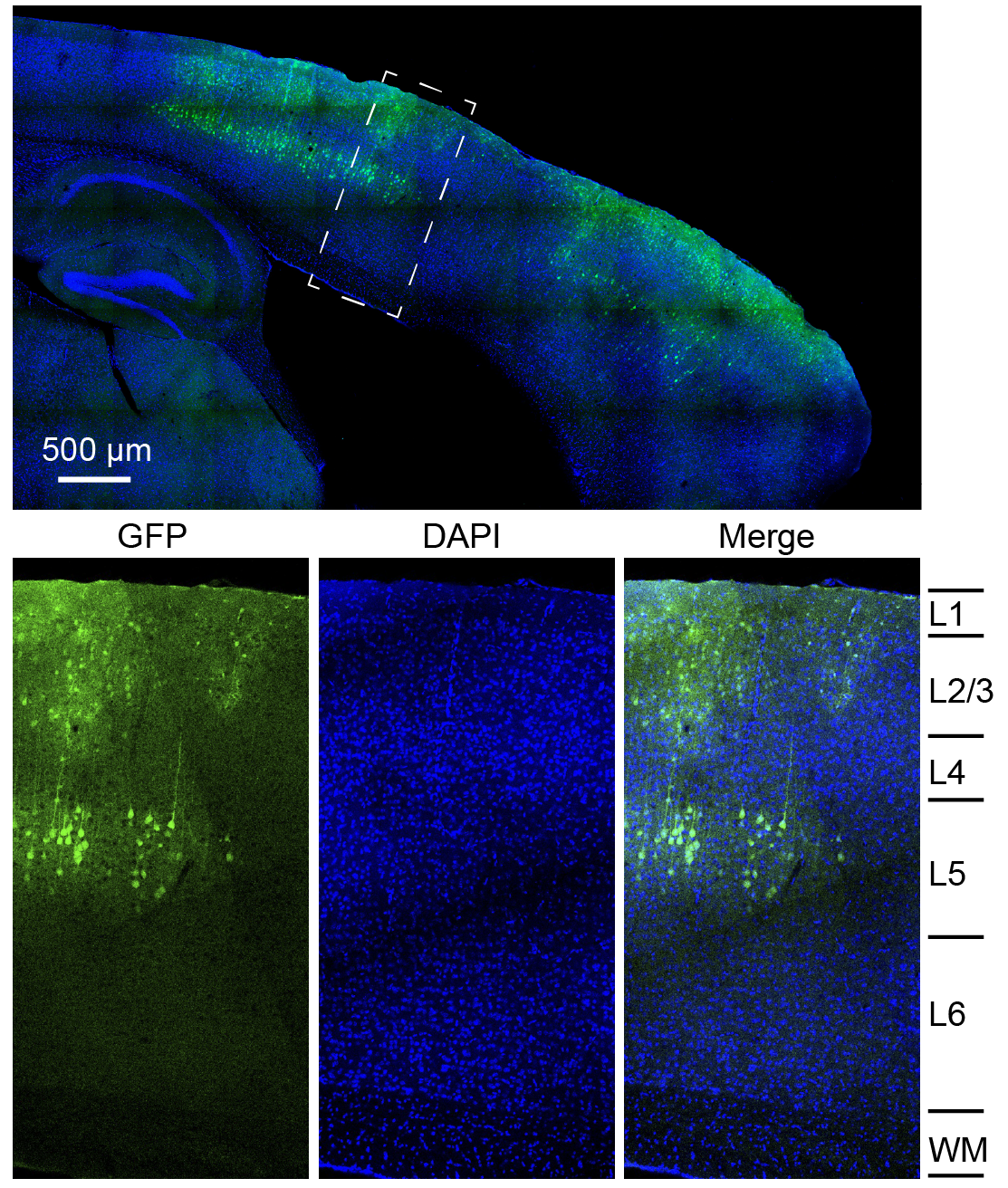

Supplement: Supplementary file 2 — Supplementary Information 2. [file 41598_2021_96332_MOESM2_ESM.tif]

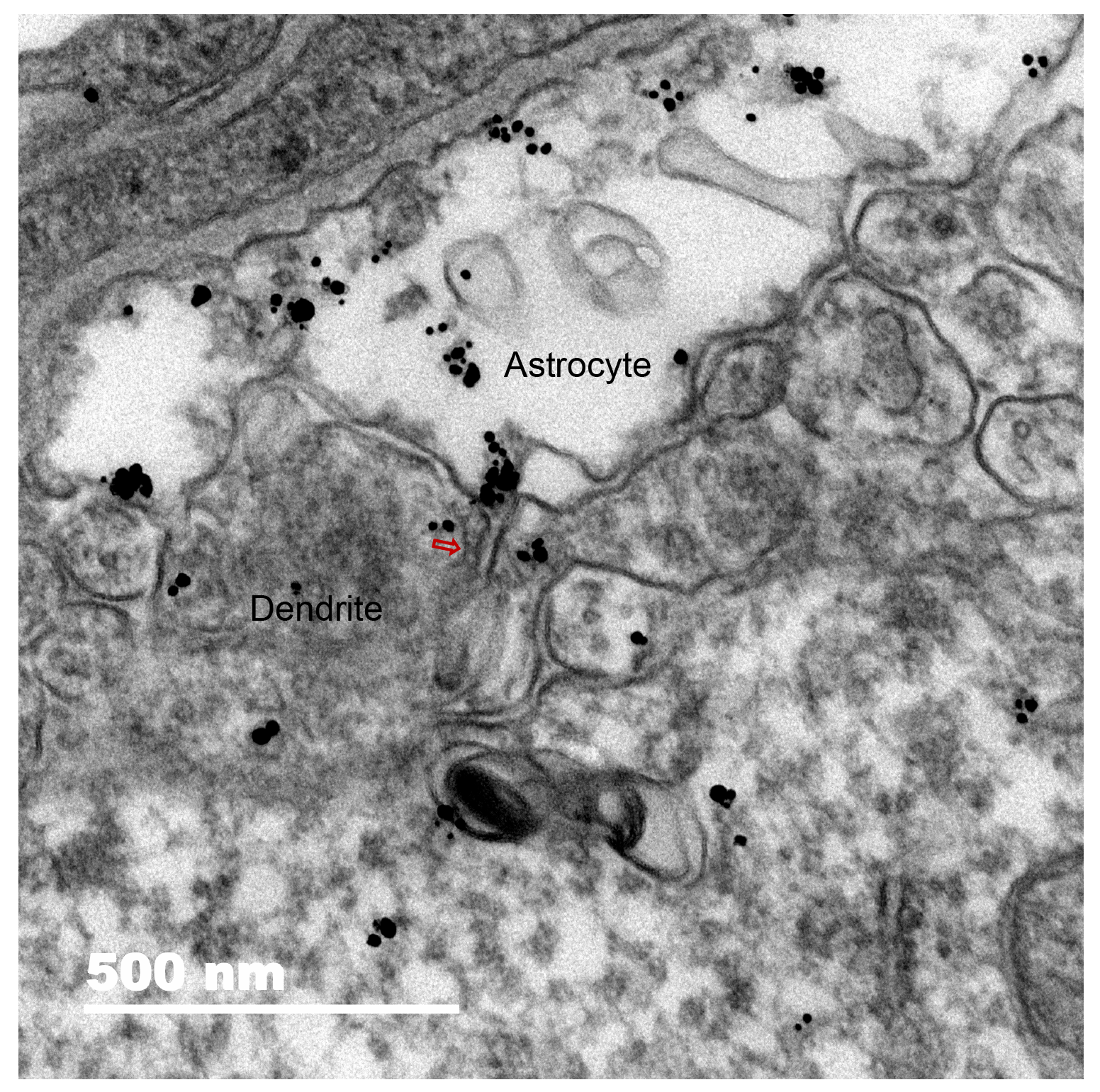

Supplement: Supplementary file 3 — Supplementary Information 3. [file 41598_2021_96332_MOESM3_ESM.tif]

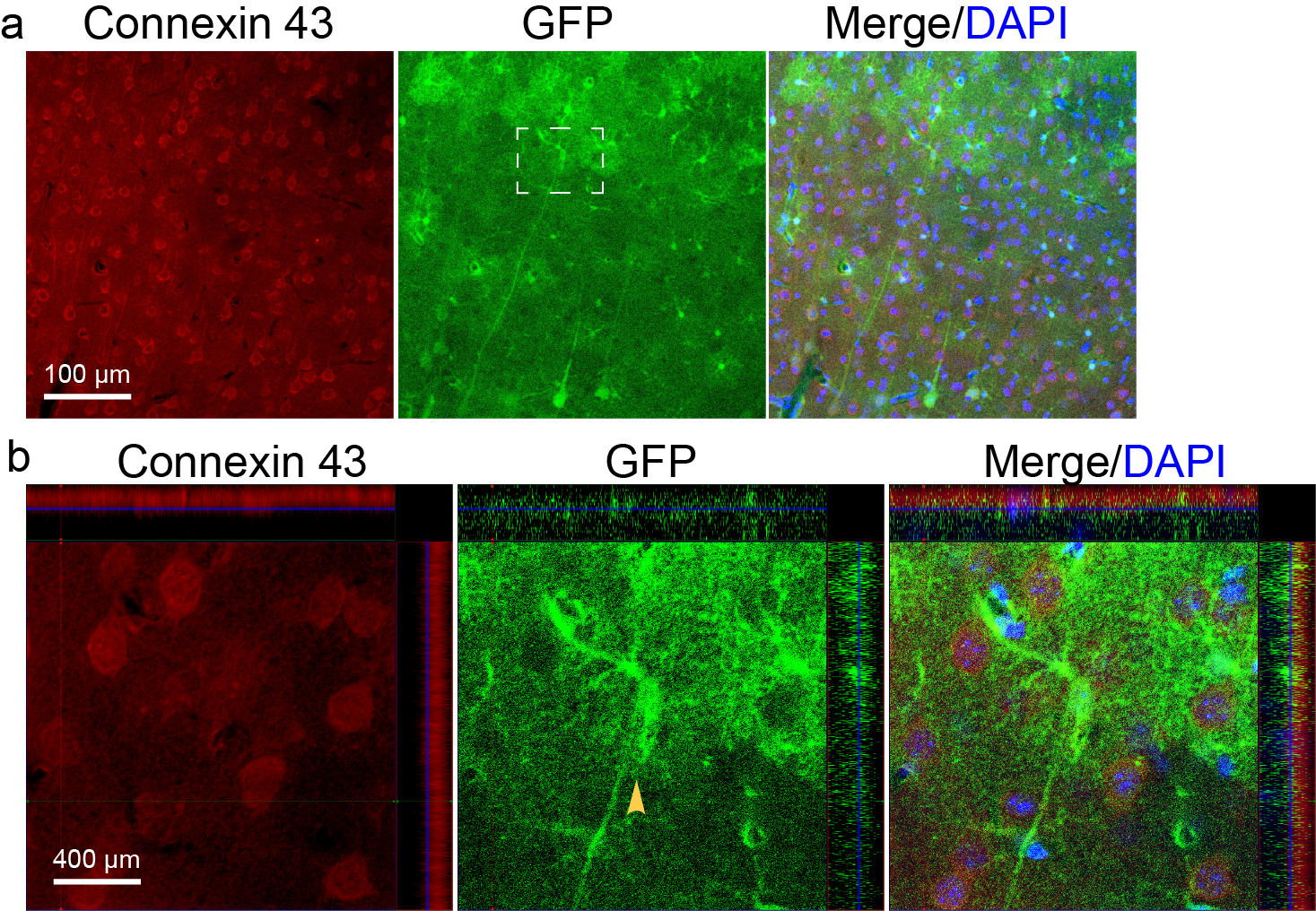

Supplement: Supplementary file 4 — Supplementary Information 4. [file 41598_2021_96332_MOESM4_ESM.tif]

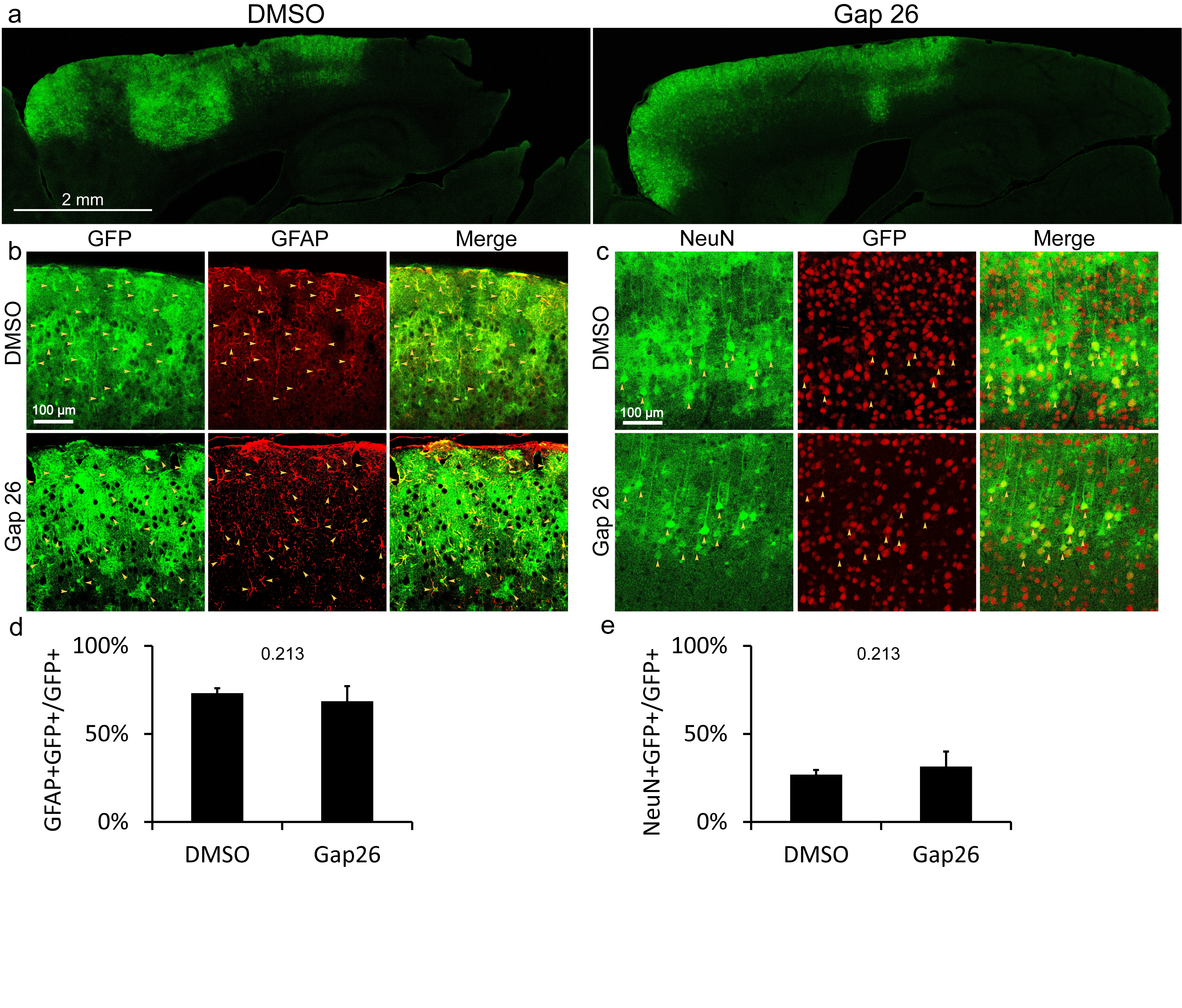

Supplement: Supplementary file 5 — Supplementary Information 5. [file 41598_2021_96332_MOESM5_ESM.tif]
