# Supplementary material for: Astrocyte-to-neuron transportation of enhanced green fluorescent protein in cerebral cortex requires F-actin dependent tunneling nanotubes
Source: Sci Rep. 2021 Aug 18;11:16798. doi: 10.1038/s41598-021-96332-5 (PMC8373867; doi:10.1038/s41598-021-96332-5)
Supplement: Supplementary file 6 — Supplementary Information 6. [file 41598_2021_96332_MOESM6_ESM.pdf]

Supplementary Figure 1. The microscopic photo with low magnification shows EGFP+ cells in cerebral cortex 5/10 days after injection of AAV.

Supplementary Figure 2. The distribution of EGFP+ cells in cerebral cortex. L, layer; WM, white matter.

Supplementary Figure 3. Detection of a TNT's ultrastructure in the cortex by immunoelectron microscopy.

Supplementary Figure 4. Immunostaning of connexin 43 and EGFP to detect the construction of TNT. (a) Low magnified image with a white rectangle to indicate the connection between an astrocyte and a neuron. (b) High magnified image of the region in the white rectangle of (a).

Supplementary Figure 5. EFGP distribution in the cortex 10 days after injecting AAV and DMSO/Gap 26. (a) The microscopic photo with low magnification shows EGFP+ cells in cerebral cortex. (b) & (c) Detection of EGFP distribution in astrocytes of layer Ⅰ-Ⅲ and neurons of layer Ⅴ at 10 DPI. Yellow arrows, double positive cells. (d) & (e) The percentage of astrocytes and neurons in EGFP+ population at 10 DPI. Statistical analysis of significance was evaluated using unpaired two-tailed t test, N = 3 independent mice.
